# A National Health and Wellness SMS Text Message Program for Breast Cancer Survivors During COVID-19 (EMPOWER-SMS COVID-19): Mixed Methods Evaluation Using the RE-AIM Framework

**DOI:** 10.2196/45164

**Published:** 2023-07-25

**Authors:** Anna C Singleton, Rebecca Raeside, Karice K Hyun, Molly Hayes, Kerry A Sherman, Elisabeth Elder, Julie Redfern, Stephanie R Partridge

**Affiliations:** 1 Engagement and Co-Design Research Hub, School of Health Sciences Faculty of Medicine and Health The University of Sydney Sydney Australia; 2 Department of Cardiology Concord Repatriation General Hospital Sydney Australia; 3 Centre for Emotional Health School of Psychological Sciences Macquarie University Sydney Australia; 4 Westmead Breast Cancer Institute Westmead Hospital Sydney Australia; 5 The George Institute for Global Health University of New South Wales Sydney Australia; 6 Research Education Network Western Sydney Local Health District Sydney Australia

**Keywords:** digital health, telemedicine, SMS text messaging, breast cancer, implementation science, cancer survivorship, supportive care, public health, COVID-19

## Abstract

**Background:**

COVID-19 lockdowns caused widespread closures of supportive care services for breast cancer survivors in Australia. In a randomized controlled trial, our team’s lifestyle-focused, evidence-based SMS text message support program (EMPOWER-SMS COVID-19) was found to be acceptable and useful for breast cancer survivors, and it was ready for rapid widespread delivery.

**Objective:**

This study aims to evaluate the reach (uptake) of an adapted 3-month lifestyle-focused SMS text message program (EMPOWER-SMS COVID-19) and barriers and enablers to implementation using the Reach, Effectiveness, Adoption, Implementation, and Maintenance (RE-AIM) framework.

**Methods:**

A mixed methods pre-post study was conducted to evaluate the EMPOWER-SMS COVID-19 program. The study evaluated the following aspects: (1) *reach/representativeness*, which refers to the proportion of participant enrollment (ie, number enrolled/number that visited the study website) and demographics (eg, age, sex, ethnicity, time since completing treatment, Index of Relative Socio-economic Advantage and Disadvantage [IRSAD; quintile 1, which refers to most disadvantaged areas, to quintile 5, which refers to least disadvantaged areas, and remoteness); (2) *effectiveness*, in which participant engagement and acceptability were evaluated using SMS text message reply data and a feedback survey (5-point Likert scale and free-text responses); (3) *adoption*, which corresponds to the proportion of organizations or health professionals who agreed to promote the program; (4) *implementation fidelity and maintenance*, which evaluated SMS text message delivery data, opt-outs, costs, and adaptations. Quantitative data were summarized using means and SDs or frequencies and percentages, while qualitative data were analyzed thematically.

**Results:**

With regard to the *reach/representativeness* of the program, 841/1340 (62.8%) participants enrolled and provided electronic consent. Participants had a mean age of 58.8 (SD 9.8; range 30-87) years. According to the data collected, most participants identified as female (837/840, 99.6%) and White (736/840, 87.6%) and nearly half (418/841, 49.7%) finished treatment ≤18 months ago. Most resided in major cities (574/838, 68.5%) and 30% (251/838) in IRSAD quintile 1 or 2. In terms of *effectiveness*, 852 replies were received from 511 unique participants (median 1; range 1-26). The most common replies were participants stating how they heard about the program (467/852, 54.8%) or “thank you” (131/852, 15.4%). None of the replies contained urgent safety concerns. Among participants who provided feedback (449/841, 53.4%), most “(strongly) agreed” the SMS text messages were easy to understand (445/448, 99.3%), useful (373/440, 84.8%), helped participants feel supported (388/448, 86.6%), and motivated participants to be physically active (312/445, 70.1%) and eat healthier (313/457, 68.5%). Free-text responses revealed 5 factors influencing engagement: (1) feeling supported and less alone, (2) motivation and reassurance for health self-management, (3) the variety of information, (4) weblinks to information and resources, and (5) the option to save the SMS text messages. Concerning *adoption*, 50% (18/36) of organizations/health professionals agreed to promote the program. With regard to implementation/maintenance, SMS text messages were delivered as planned (97.43% [41,257/42,344] of SMS text messages were successfully delivered) with minimal opt-outs (62/838, 7.4%) and low cost (Aus $15.40/participant; Aus $1=US $0.67). No adaptations were made during the intervention period. Postintervention adaptations included adding weblinks and participant-selected customizations.

**Conclusions:**

EMPOWER-SMS COVID-19 was implemented quickly, had a broad reach, and had high engagement and acceptability among socioeconomically diverse participants. The program had high fidelity, low cost, and required minimal staff oversight, which may facilitate future implementation. However, further research is needed to evaluate barriers and enablers to adoption and implementation for health professionals and strategies for long-term maintenance.

## Introduction

Globally, the 5-year survival rates for early stage breast cancer in high-income countries vary between 80% and 90% [1]. In Australia, the 5-year survival rates for breast cancer are reported to be 91%; additionally, it is estimated that over 200,000 Australians have completed active breast cancer treatment, including surgery, chemotherapy, and radiotherapy, in the past 10 years [2]. Posttreatment guidelines encourage breast cancer survivors to engage in regular physical activity (~150 minutes per week of moderate-intensity activity), maintain a healthy weight, eat a healthy diet, and if prescribed, adhere to medication to reduce the risks of breast cancer recurrence [3]. In-person health support has been found effective for improving mental and physical health, quality of life, and symptom management for breast cancer survivors [4-7]. However, personal and environmental barriers, including ongoing physical (eg, lymphoedema, fatigue) [8,9] and mental health challenges (eg, anxiety, depression) [10], financial stresses, and location and timing of programs, make it challenging to attend in-person programs [11]. In March 2020, the World Health Organization declared the global novel COVID-19 outbreak as a pandemic [12], which caused widespread cancellations to in-person cancer services globally [13,14]. In Australia, new public health safety measures were implemented, including maintaining 2-m physical distance between people and limiting group activities, causing further disruptions to survivorship care [15]. There was an urgent need for an accessible health support strategy.

Studies [16,17] suggest that over three-quarters of people worldwide own a mobile phone, and in Australia, 90% of adults own a mobile phone. Delivering health support via mobile phone apps or SMS text messages (ie, mobile health [mHealth]) has become ubiquitous. Mobile phone services reach 99% of the Australian population, including rural and remote communities, compared with only 91% having internet access. SMS text messages are therefore the most accessible mobile health technology because they are inexpensive to send, free to receive, and do not require a smartphone or internet connection. Research shows that SMS text messages are an effective and acceptable way to deliver health support to patients with chronic diseases [18-23], including those with breast cancer [24-27]. Recent evidence suggests that an SMS text message intervention was effective in providing support for individuals experiencing stress and anxiety during the COVID-19 pandemic in Canada [28,29]. However, evaluating the widespread implementation of a community-based, readily available SMS text message intervention to support Australian breast cancer survivors’ health and wellness during the COVID-19 lockdowns had not been explored.

Using the enhanced Reach, Effectiveness, Adoption, Implementation, and Maintenance (RE-AIM) framework [30], this study aimed to evaluate the nationwide implementation of a 3-month pre-post SMS text message intervention called “EMPOWER-SMS COVID-19” to support breast cancer survivors’ health and wellness during the COVID-19 pandemic in Australia. The program is based on our team’s EMPOWER-SMS randomized controlled trial [24,25,31], which found a 6-month SMS text message intervention acceptable and useful for supporting breast cancer survivors’ health and wellness [24,25]. We hypothesized that the adapted 3-month EMPOWER-SMS COVID-19 program would have widespread uptake and be perceived as useful, acceptable, and motivating for participants.

## Methods

### Study Design

A mixed methods evaluation of a 3-month pre-post SMS text message support program for breast cancer survivors during the COVID-19 pandemic was conducted following the enhanced RE-AIM framework [30]. This study is reported according to the TIDieR (Template for Intervention Description and Replication) checklist (Multimedia Appendix 1) [32].

### Ethics Approval

The study was approved by the University of Sydney Human Research Ethics Committee (Approval number: 2020/181) and all participants provided informed electronic consent (e-consent). All procedures performed in studies involving human participants were in accordance with the ethical standards of the institutional or national research committee and with the 1964 Helsinki declaration and its later amendments or comparable ethical standards.

### Participants and Recruitment

Participants were eligible if they (1) completed active breast cancer treatments (no time limitation), including surgery or chemotherapy or radiotherapy, although they could still be taking endocrine therapy tablets; (2) had an Australian mobile phone number; and (3) had sufficient English skills to provide informed e-consent. Participants were recruited from April 29, 2020, to February 10, 2021, through (1) paid and unpaid advertisements on social media (eg, Facebook, Instagram, and Twitter); (2) emails from breast cancer organizations (eg, McGrath Foundation, Cancer Council New South Wales, and Breast Cancer Network Australia), including the National Breast Cancer Foundation’s “Register4 Research for Cancer” national online registry of people interested in participating in research; and (3) radio advertisements. Participants could access the study registration website either through a secure weblink or by texting a keyword (wellness) to the study mobile phone number, which would automatically deliver a reply SMS text message containing a secure weblink or by scanning a QR code. The registration website was hosted on the University of Sydney–approved Research Electronic Data Capture (REDCap) application and contained the participant information sheet and e-consent form. Next, participants provided demographic data (ie, sex, age, postcode, ethnic background, and time since completing treatment). To complete enrollment, participants entered their preferred name (nickname) and mobile number into a web-based automated SMS text message delivery software (Burst SMS). The software allowed web-based tracking of SMS text message delivery (eg, successfully or unsuccessfully delivered) and participant replies, and enables researchers to respond to participants, if needed.

### EMPOWER-SMS COVID-19 Intervention

The 3-month EMPOWER-SMS COVID-19 intervention was adapted from the original 6-month EMPOWER-SMS intervention [24,25,31], which was co-designed by breast cancer survivors, health professionals, and researchers with expertise in oncology, dietetics, psychology, physiotherapy, and public health [33]. The original SMS text message content themes included social and emotional support, physical activity, healthy eating, and general breast cancer information. The main changes were that (1) the program was shortened to 3 months, because at the time of study design, it was unknown how long the pandemic and associated lockdowns would continue in Australia; (2) 12 SMS text messages were added regarding general and breast cancer–specific advice for managing COVID-19 and lockdowns; (3) all text SMS text message content was edited to encourage COVID-19 safety regulations in Australia, such as abiding by social distancing (remaining 1.5 m away from other people) and quarantine; and (4) rather than including a hospital-specific sign-off at the end of each SMS text message, such as “from the Breast Cancer Institute team,” no sign-off was included. Example SMS text messages from each content theme are presented in Multimedia Appendix 2. SMS text messages were evidence based, semipersonalized using participants’ “preferred name” or “nickname” and positively toned [33]. Based on the original EMPOWER-SMS co-design data and our team’s previous qualitative research regarding the timing of SMS text message delivery, EMPOWER-SMS COVID-19 delivered 4 SMS text messages per week (free of charge) at random times between 9 AM and 6 PM, on random days for 3 months [19,20,33].

Unlike the original program, which was 1 way (ie, no replies), participants in EMPOWER-SMS COVID-19 were informed that they could reply and receive a response within 72 hours. Only 1 SMS text message asked a direct question, which was about how participants heard about the program, with the following options: (1) health professional, (2) Facebook, (3) Instagram, (4) breast cancer organization, and (5) other (please specify). The final SMS text message included a secure weblink to a user feedback survey regarding the acceptability and utility of the program (described in detail later). After 1 month, if participants had not completed the survey, this SMS text message was re-sent by the research team. For safety, a member of the research team monitored replies and responded when appropriate. Participants could opt-out at any time by replying “STOP” or requesting to unsubscribe and they were removed from the program within 72 hours.

### RE-AIM Framework

*Reach and representativeness* was evaluated using the percentage of people who clicked on the study website versus people who enrolled in the study (number enrolled/number that clicked study website × 100) and participant demographics, including age (years), ethnicity, sex (male, female, or prefer not to say), time since completing treatment (<6 months, 6-18 months ago, 18 months to 3 years ago, and >3 years ago), and postcode. Postcode was used to determine participants’ Index of Relative Socio-economic Advantage and Disadvantage (IRSAD) [34], which groups postcodes into quintiles from 1 (most disadvantaged area) to 5 (least disadvantaged area). Postcode was also used to determine the participants’ remoteness using the Australian Statistical Geography Standard of Remoteness Areas [35], categorized as living in a Major City, Inner Regional Australia, Outer Regional Australia, Remote Australia, and Very Remote Australia.

*Effectiveness* for changing a specific outcome was not evaluated in this study given the previous evaluation of EMPOWER-SMS in a randomized controlled trial [24,25]. However, participant engagement and satisfaction with the EMPOWER-SMS COVID-19 intervention were evaluated based on (1) *SMS text message reply data* (the number and content of SMS text message replies, including text, emojis [a small digital image or icon used to express an idea or emotion), or “reactions,” which occur when a participant clicks an SMS text message and clicks “like,” “love,” or “emphasize”; and (2) *user feedback survey*, a researcher-designed survey, which has been used in previous studies [19,25,36], that contained 13 questions, including 11 five-point Likert scale questions (1=strongly disagree to 5=strongly agree) regarding the perceived usefulness of the program (ease of understanding and supportiveness); participants’ motivation to manage their health (eg, eating healthier or being physically active); whether participants shared the program with family, friends, or medical professionals; and participants’ preferences for program characteristics, including the number of SMS text messages per week, the time of the day these SMS text messages were received, the length of the program, and the formality of the language within the SMS text messages. The survey additionally incorporated 2 free-text questions where participants were asked to provide their thoughts on what they liked the most and least about the program, as well as any suggestions for improvement.

As this intervention was delivered virtually via a study website, *adoption* was evaluated at the setting level based on the proportion of organizations or health professionals who agreed to promote the study website and reasons for nonpromotion. A variety of breast cancer organizations were approached via email, including not-for-profit community-based organizations, hospital-based organisations, and individual health professionals. An email log was kept to confirm promotions or rejections. Confirmation of study promotion was also estimated using participant SMS text message reply data for “How you heard about the program,” with the following response options: (1) health professional, (2) Facebook, (3) Instagram, (4) breast cancer organization, and (5) other (please specify).

*Implementation and maintenance* was evaluated by the fidelity of the intervention including (1) *SMS text message delivery data* (April 29, 2020, to May 10, 2021), which refers to the number of SMS text messages successfully delivered or unsuccessfully delivered (bounced); (2) the percentage of opt-outs (number of opt-outs/number at baseline × 100); (3) the direct intervention costs, including delivering the SMS text messages, monthly fees for the automated SMS text message delivery software and advertising fees; (4) the cost of staff time to monitor and respond to incoming SMS text messages, at an annual salary of Aus $78,760 (Aus $43.27/hour; Aus $1=US $0.67); and (5) adaptations made to the intervention.

### Data Analyses

Categorical data, including the SMS text message delivery data and quantitative feedback survey data, were summarized by frequencies and percentages and continuous data were summarized as means and SDs. Demographic characteristics of patients who enrolled and opted out were compared using independent *t* test (unpaired, 2-tailed) and Fisher exact test. To evaluate barriers and enablers to participant engagement, SMS text message responses and free-text feedback survey responses were independently coded into themes using the framework approach. Two researchers (RR and ACS) familiarized themselves with the data and independently inductively coded the data into themes and subthemes, which were then repeatedly compared and adapted until final themes and subthemes were established. Any disagreements were reviewed by a third reviewer (JR) and discussed until an agreement was reached [37].

## Results

### Overview

Within 6 weeks of the Australian COVID-19 lockdown that began on March 23, 2020, EMPOWER-SMS was adapted to EMPOWER-SMS COVID-19. Ethics and legal approvals were obtained, and the automated SMS text message software was set up for nationwide implementation.

### Reach

**Figure 1:**
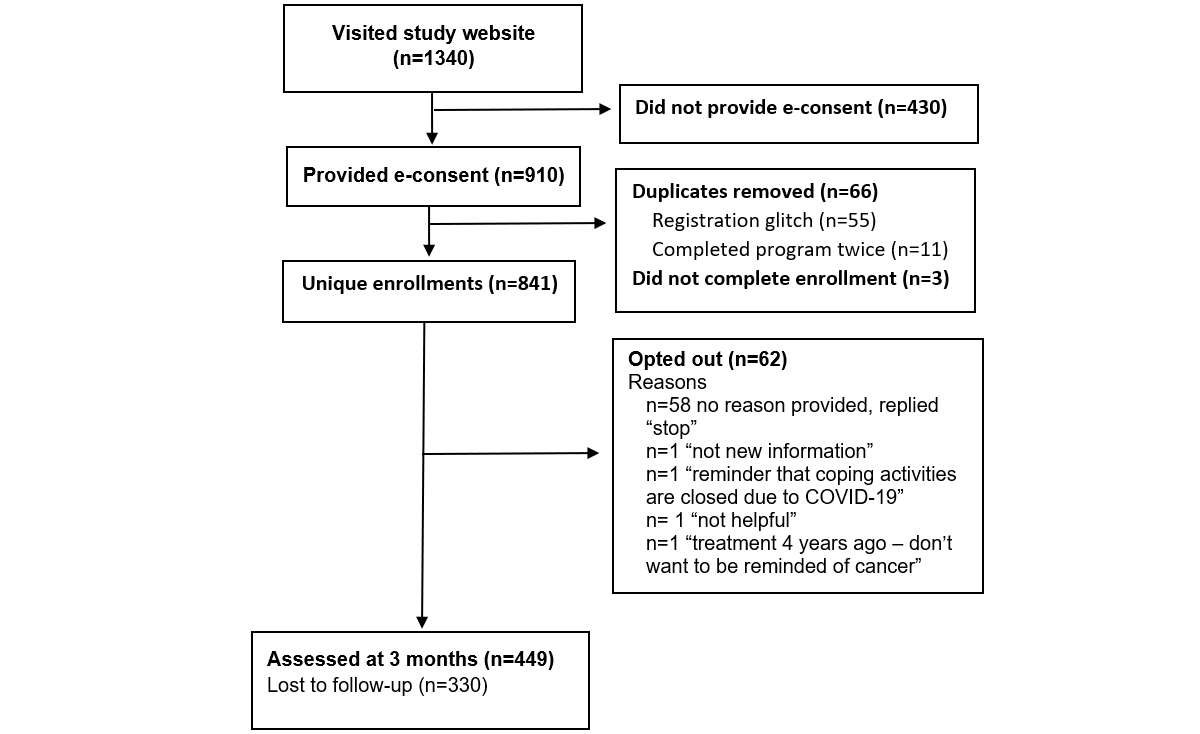
Study flow diagram. e-consent: electronic consent.

A total of 1340 individuals visited the study registration website to read the electronic participant information sheet; 852 participants provided e-consent, of which 841 completed the demographic data and enrolled in EMPOWER-SMS COVID-19 (enrollment rate: 841/1340, 62.8%; Figure 1). Participants had a mean age of 58.8 (SD 9.8) years (range 30-87 years). Most participants identified as female (837/840, 99.6%) and White (736/840, 87.6%; Table 1). Half of the participants finished treatment within the past 18 months (418/841, 49.7%) and 301/841 (35.8%) finished treatment more than 3 years ago. Participants enrolled from all states and territories in Australia: New South Wales (332/841, 39.5%), Victoria (175/841, 20.8%), Queensland (143/841, 17.0%), South Australia (65/841, 7.7%), Western Australia (69/841, 8.2%), Australian Capital Territory (28/841, 3.3%), Tasmania (25/841, 2.9%), and Northern Territory (4/841, 0.5%). Based on participants’ postcode, 261/838 (31.1%) resided in Australia’s least disadvantaged areas (IRSAD quintile 5) and 101/838 (12.1%) participants resided in Australia’s most disadvantaged areas (quintile 1). Most participants lived in Major Cities (574/838, 68.5%) followed by Inner or Outer Regional (255/838, 30.4%) and Remote or Very Remote (9/838, 1.1%).

**Table 1:** Baseline participant characteristics (N=841).

Characteristics		Values, n/N (%)
**Demographics**		
Age (years), mean (SD); range		58.8 (9.8); 30-87
**Sex**		
	Male	2/840 (0.2)
	Female	837/840 (99.6)
	Prefer not to say	1/840 (0.1)
**Ethnicity**		
	Aboriginal or Torres Strait Islander	7/840 (0.8)
	New Zealander or Māori	15/840 (1.8)
	White	736/840 (87.6)
	Chinese	5/840 (0.6)
	South Asian (Bangladesh, India, Nepal, Pakistan, or Sri Lanka)	9/840 (1.1)
	Asian (excluding South Asian)	13/840 (1.5)
	Middle Eastern	5/840 (0.6)
	South American	3/840 (0.4)
	Other	38/840 (4.5)
	Prefer not to say	9/840 (1.1)
**Time since finishing active treatment**		
	Within the last 6 months	266/841 (31.6)
	Within the last 6-18 months	152/841 (18.1)
	Within the last 18 months-3 years	107/841 (12.7)
	3 or more years ago	301/841 (35.8)
	Prefer not to say	15/841 (1.8)
**Index of Relative Socio-economic Advantage and Disadvantage, categorized from postcode**		
	Quintile 1 (most disadvantaged)	101/838 (12.1)
	Quintile 2	150/838 (17.9)
	Quintile 3	176/838 (21.0)
	Quintile 4	150/838 (17.9)
	Quintile 5 (least disadvantaged)	261/838 (31.1)
**Australian standard geographical standard remoteness area**		
	Major City	574/838 (68.5)
	Inner Regional Australia	193/838 (23.0)
	Outer Regional Australia	62/838 (7.4)
	Remote Australia	6/838 (0.7)
	Very Remote Australia	3/838 (0.4)

### Effectiveness

*SMS text message reply data* revealed frequent participant engagement, with 852 SMS text message replies from 511 unique participants (median number of replies per participant 1; range 1-26). The most common themes within the replies were “how they heard about the program” (467/852, 54.8%), “thank you” (131/852, 15.4%), simple replies including “Ok,” “Definitely,” “Yes,” or emojis (85/852, 10.0%), sharing personal experiences (53/852, 6.2%), “loved” or “liked” reactions (44/852, 5.2%), and questions about an SMS text message (14/852, 1.6%; see examples in Textbox 1).

**SMS text message reply themes and example quotations.**

1. **Thank you**
  - *Thank you. I need to hear this sometimes xx* [Age 49, Major City, finished treatment <6 months ago]
  - *Thank you so much for your information and help, all ongoing but doing best I can, you all take care*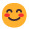 [Age 68, Major City, finished treatment 6-18 months ago]
  - *Thank you, your information and hints have been helpful* [Age 75, Major City, finished treatment ≥3 years ago]
  - *Thankyou for the texts. I’ve appreciated them even though my journey began 10 years ago.* [Age 65, Inner Regional Australia, finished treatment ≥3 years ago]
2. **Emojis and Simple Responses**
  - *Definitely* [Age 76, Major City, finished treatment ≥3 years ago]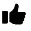 [Age 55, Inner Regional Australia, finished treatment 18 months-3 years ago]
  - *Will do*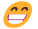 [Age 54, finished treatment ≥3 years ago, Major City]
3. **Sharing Personal Experiences**
  - *I have just enjoyed a lovely morning with a friend I met when we were both having radiation treatment after breast cancer surgery in early 2002. We are both still here!!* [Age 75, Inner Regional Australia, finished treatment ≥3 years ago]
  - *Yes I walked the dogs* [Age 66, Major City, finished treatment ≥3 years ago]
  - *Great idea - sitting too long makes it difficult for me to walk straight away - need to stand in the one spot to gain my composure.* [Age 74, Outer Regional Australia, finished treatment with 18 months-3 years]
  - *Mainly when I’m working. Usually I take a small break and get away from my computer.* [Age 66, Major City, finished treatment ≥3 years ago]
  - *I am going to the pool for an hour most mornings. Easy exercise but very tiring.* [Age 76, Inner Regional Australia, finished treatment with 18 months-3 years]
  - *Yes, I did my 1 hr walk with a friend! Yesterday I had Taichi, Tues and Wed walked and Monday was pool session.*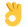 [Age 69, Inner Regional Australia, finished treatment ≥3 years ago]
4. **Asking Questions**
  - *Not exercise but doing hectic housework clearing the junk in my bedroom, What I did was go and stop when I feel exhausted then go again and stop do you think it’s ok that way?* [Age 77, Major City, finished treatment within the last 6-18 months]
  - *Can you put me in touch with someone who has been through the AC-TH chemo regimen for HER2 positive breast cancer? Thanks so much*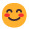 [Age 65, Inner Regional Australia, prefer not to say when finished treatment]
  - *What about arthritis, sensitivity caused by treatment. Where do we go for help with that?* [Age 74, Major City, finished treatment within the last 6 months]
5. **Sharing Gratitude Lists (Three Things They Are Grateful for Today)**
  - *Grateful for a thoughtful husband: grateful for a sunny day, grateful for my minimal needs* [Age 80, Major City, finished treatment within the last 6 months]
  - *I am grateful to be healthy and active. I am grateful for my family as my diagnosis was awful for them too. I am grateful to be working from home during COVID* [Age 64, Major City, finished treatment within the last 18 months-3 years]
  - *My daughter, my dog and my dad.*[Age 54, Inner Regional Australia, finished treatment ≥3 years ago]

A total of 449/841 (53.4%) participants completed the user feedback survey (Figure 1), with the responses summarized in Table 2. Overall, participants were highly satisfied with the intervention; participants “agreed” or “strongly agreed” that the SMS text messages were easy to understand (445/448, 99.3%), helped them feel supported (388/448, 86.6%), were useful (373/440, 84.8%), and helped them manage their health (311/448, 69.4%).

These quantitative results were complemented by participants’ qualitative free-text feedback. For example, the thematic analysis revealed that the positivity of the SMS text message content was a prominent theme:

Despite having a great support network, I found that on the occasional day when feeling down/lonely/anxious the messages gave a real boost that someone out there cares enough to send a message which was positive and uplifting. I found myself looking forward to them.

Age 47, Outer Regional Australia, finished treatment within the last 6-18 months

Someone was reaching out & some days it was just what I needed. Some of the websites really helped, but the positive words were the best.

Age 63, Major City, finished treatment within the last 6 months

Participants also expressed that they enjoyed the overall program, rather than 1 specific type of SMS text message:

Look it was nothing in particular just lots of handy hits to help me through my days when I was feeling a bit down about having breast cancer. 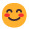

Age 60, Major City, finished treatment within the last 6 months

Two-thirds of the survey participants felt the program length was “just right” but one-third felt that 3 months was too short and wanted to be given an option to continue. This was supported by the thematic analysis, where “choice” emerged as a theme:

The program should be ongoing and the patient should be given choice when they want to cancel the subscription.

Age 45, Major City, finished treatment within the last months

Survey results also revealed that some participants shared the SMS text messages with family, friends, or other people diagnosed with cancer. Qualitative results revealed that sharing was aimed at providing information or support. For example, 1 participant said:

I shared the messages with a friend who was going through chemo and it has given her a boost too.

Age 51, Major City, finished treatment ≥3 years ago

**Table 2:** Perceived acceptability and usefulness of the EMPOWER-SMS COVID-19 program.

Characteristic			Value, n/N (%)
**Usefulness^a^**			
	Found the SMS text messages useful		373/440 (84.8)
	Majority of the SMS text messages were easy to understand		445/448 (99.3)
	Program helped participant feel supported		388/448 (86.6)
**Motivation and health management^a^**			
		The SMS text messages helped me manage my health	311/448 (69.4)
		The SMS text messages motivated me to be physically active	312/445 (70.1)
		The SMS text messages motivated to eat healthier	313/457 (68.5)
**SMS text message sharing**			
		Friend	102/449 (22.7)
		Family member	146/449 (32.5)
		General practitioner/doctor	13/449 (2.9)
		Specialist	8/449 (1.8)
		Nobody	239/449 (53.2)
**Program characteristics**			
		Language of the SMS text messages was “just right”^b^	424/447 (94.9)
		Number of SMS text messages per week was “just right”^c^	373/449 (83.1)
		The 3-month program length was “just right”^d^	288/446 (64.6)
		The 3-month program length was “too short” or “much too short”^d^	138/446 (30.9)
		Time of the day receiving the SMS text messages was appropriate	356/445 (80.0)

^a^Response options were “strongly disagree, disagree, neutral, agree, strongly agree.” The proportion of participants who “strongly agree” and “agree” is reported.

^b^Response options were “too casual, casual, just right, formal, too formal.”

^c^Response options were “much too many, too many, just right, too few, much too few.”

^d^Response options were “much too long, too long, just right, too short, much too short.”

However, few participants shared the SMS text messages with a medical professional (general practitioner or specialist) and one-half did not share the SMS text messages with anyone. Qualitative results revealed that participants were keeping the program as a personal experience, “just for me”:

I like that it was something for me that l had no need to discuss with others.

Age 62, Major City, finished treatment ≥3 years ago

Felt connected, cared about without feeling a burden to anyone close.

Age 60, Inner Regional Australia, finished treatment ≥3 years ago

The thematic analysis also revealed several factors influencing participants’ engagement with the program, including (1) continuity of supportive care, (2) having motivation and reassurance for health self-management, (3) the variety of information, (4) weblinks to information and resources, and (5) the option to save the SMS text messages (Textbox 2). Participants stressed that this was of particular importance during the uncertainty of the COVID-19 lockdowns and legislation changes:

Contact with someone else was great and the content made me feel normal – very helpful in an isolated environment.

Age 62, Major City, finished treatment within the last 6-18 months

It just made me feel that I wasn’t alone especially when most services were cut off! It was just a gentle reminder to look after myself and that I was important.

Age 56, Major City, finished treatment within the last 6 months

**Enablers for participants’ engagement with EMPOWER-SMS COVID-19 and suggestions for improvement.**

**Theme 1: Continuity of Supportive Care**

- Feeling supported

It made me feel supported and that someone cared that I am still not well (everyone expects you to be better a couple of weeks after chemo) some messages were just the right thing at the right time.

Age 52, Major City, finished treatment within the last 6-18 months

Very timely, our support group was cancelled due to COVID-19, it was nice to received even a text message, I felt supported.

Age 48, Major City, finished treatment ≥3 years ago

Sometimes a cancer diagnosis can be isolating, it is great to feel supported

Age 62, Major City, finished treatment ≥3 years ago

It reminded me that there was someone thinking of my wellbeing

Age 50, Remote Australia, finished within the last 6 months

- Feeling less alone

Reminded me I wasn’t alone in dealing with cancer and it’s fallout, that many others are also dealing with it.

Age 56, Major City, finished treatment within the last 6-18 months

I looked forward to seeing the messages. It made me feel more connected as I live alone :)

Age 70, Outer Regional Australia, finished treatment ≥3 years ago

Even though I am 11 years post BC I struggle everyday as my life has completely changed - these messages helped me feel not so isolated and alone.

Age 64, Inner Regional Australia, finished treatment ≥3 years ago

**Theme 2: Motivation and Reassurance for Health Self-Management**

- Gentle health reminders

Reminders to take time out for myself during the day, and importance of eating well and exercising

Age 44, Outer Regional Australia, finished treatment ≥3 years ago

They were reminders about aspects of health and wellbeing that were simple, easy to follow and not too formal in tone.

Age 60, Major City, finished treatment ≥3 years ago

I liked that the messages gently reminded me to take care of 'me' during the busyness in my life.

Age 54, Major City, finished treatment within the last 6-18 months

- Motivation to change behaviors

I loved receiving the messages, it has really helped me to prioritise myself and do small things each day for my recovery.

Age 49, Inner Regional Australia, finished treatment within the last 6 months

It helped reinforce things I should be doing. No one else knew that I changed my behaviour because I was told in a message, I just looked like I was doing it on my own

Age 40, Outer Regional Australia, finished treatment within the last 6 months

I just liked getting a little nudge to stop and think how I was going, and if I needed to do or change something to increase my wellbeing

Age 44, Major City, finished treatment within the last 6 months

- Reassurance of doing the right things

The messages confirmed that I am on the right track

Age 71, Inner Regional Australia, finished treatment ≥3 years ago

It made me feel like I was supported, even though I didn't really learn anything new. I come from a nursing background […] but it did give me confidence that what I knew already was correct.

Age 58, Major City, finished treatment within the last 6 months

**Theme 3: Variety of Information**

Wide variety of useful information - felt very friendly & personal

Age 67, Inner Regional Australia, finished treatment within the last 6-18 months

The variety of suggestions that were relevant with dealing with a cancer diagnosis, treatment side effects and range of effects during and the aftermath and ongoing day to day living; emotionally, socially, physically, sensory and sexually!!

Age 61, Major City, finished treatment within the last 6-18 months

I liked the variety of themes but especially mental health re[garding] covid

Age 55, Inner Regional Australia, finished treatment within the last 6-18 months

**Theme 4: Weblinks to Information and Resources**

Links to more in-depth information. Found very helpful.

Age 66, Major City, finished treatment ≥3 years ago

Great size messages and useful links. Quick and easy to access

Age 51, Major City, finished treatment ≥3 years ago

**Theme 5: Option to Save the SMS Text Messages**

I liked being able to scroll back through the messages at times when I felt that I needed support.

Age 58, Major City, finished treatment ≥3 years ago

**Suggestions for Improvement: More Personalization and Choice**

- Control Over Program Structure
- Program Length

I would have loved to see it continue until I decided to stop it rather than it being stopped. I'll miss it :-(

Age 47, Outer Regional Australia, finished treatment within the last 6-18 months

It was too short. I am saying this because to change my mindset to do some of the things suggested would take longer. When you first read the message, you think that's good/not so good idea. But it needs to be reinforced a bit (even if some of the messages were repeated.)

Age 72, Major City, finished treatment within the last 6 months

Keep it going for 6 months even 12 months. We are still really vulnerable for a long time post active treatment and these messages were like my silent friend.

Age 47, Major City, finished treatment within the last 6 months

- Frequency of SMS text message delivery

I wonder if it would be possible to tailor frequency of messages and length of program to roughly where someone is up to. More frequent for longer for those closer to active treatment, less frequent for less time for someone further out. Approaching 5 years from completing treatment, I valued this, but would have got far more benefit in the first couple of years

Age 54, Major City, finished treatment ≥3 years ago

Too many messages. Maybe a set time 3 times a week or consider consistency based on requirements or experience

Age 47, Major City, finished treatment within the last 6-18 months

- Time of the day

Texts arriving in the middle of the workday didn't allow me to really take in the content. Would have preferred they be sent at night around 8pm.

Age 44, Outer Regional Australia, finished treatment ≥3 years ago

- More specific SMS Text message content

Some messages too generic to participant journey. A more targeted approach would be more appropriate when the program is released to all patients

Age 69, Major City, finished treatment within the last 6-18 months

A little bit broad, perhaps being a little more specific re[garding] exercise, eating etc.

Age 55, Inner Regional Australia, finished treatment ≥3 years ago

- SMS text messages personalized for people who finished treatment 3+ years ago

I am 4 years post diagnosis so messages around keeping healthy and positive were more beneficial

Age 49, Major City, finished treatment ≥3 years ago

Based on the quantitative and qualitative feedback survey results, there were few barriers to participant engagement. However, some participants would have preferred control over the program structure, including the program length, frequency of SMS text message delivery, and the time-of-the-day SMS text messages were delivered. Participants suggested the option to control these factors to best suit individuals’ preferences, schedules, and lifestyle (Textbox 2). The second barrier was that the SMS text message content was too general for some participants and they felt that the content was not new or relevant to them, especially those who completed treatment more than 3 years ago. Participants suggested adding more links to “in-depth” information, especially mental health, diet, and exercise and having different SMS text messages for people during treatment, immediately after treatment, and years after finishing treatment.

Maybe add some links to relevant research or in-depth discussion on the topic presented

Age 67, Major City, finished treatment ≥3 years ago

It would be great to see something like this rolled out whilst undergoing treatment. As sometimes the online networks can be overwhelming and depressing when you read the sad stories.

Age 49, Major City, finished treatment within the last 6 months

I’m 7 years out from diagnosis. When I’m feeling good, I don’t like being reminded about Breast Cancer. […] I absolutely LOVED getting messages about exercise, mindfulness, healthy eating etc. The type of “healthy message” I might get even if I didn’t have cancer. Perhaps there could be an option to eliminate cancer references for those who just want healthy lifestyle messages?

Age 58, Major city, finished treatment ≥3 years ago

Other participants found it acceptable to receive some SMS text messages that were not directly relevant to them:

Some of the messages weren’t personally relevant, but that is to be expected. I just ignored those I felt weren’t for me.

Age 61, Outer Regional Australia, finished treatment within the last 18-months to 3 years

Suggestions were also made by participants to expand the program to other types of cancers, to have a program for carers or families of cancer survivors.

Just continue to provide to all breast cancer patients and expand the program to all of those who experience a cancer diagnoses as well as their supports/carers!! More work but it is an excellent resource for all!

Age 61, Major City, finished treatment within the last 6-18 months

### Adoption

A total of 36 organizations and health professionals were emailed, including 28 not-for-profit community-based organizations, 3 hospital-based organizations, and 5 health professionals. Of these, 18 (50%) agreed to promote EMPOWER-SMS COVID-19. One-half (14/28) of the not-for-profit organizations agreed to promote the study website via e-newsletter, email, or social media, including Australian’s largest breast cancer organizations, the McGrath Foundation and Breast Cancer Network Australia, and Cancer Council New South Wales. Two (2/3, 67%) hospital-based organisations, the Westmead Breast Cancer Institute and Sydney Survivorship Centre, agreed to promote EMPOWER-SMS COVID-19 via email or as a dedicated page on their website. Two (2/5, 40%) health professionals agreed to promote the program via social media and word of mouth. Of those who did not promote the program, 17/18 (94%) did not reply to our email and 1/18 (6%), a not-for-profit community-based organization in Western Australia, indicated that their members received enough information and did not need additional support. A total of 461/841 (54.8%) participants replied to our question regarding how they heard about the program, with most responding with social media (246/461, 53.4%), including Facebook (225/461, 48.8%) and Instagram (21/461, 4.6%), or a breast cancer organization (170/461, 36.9%), and some through a health care professional (5/461, 1.1%) or “other” (29/461, 6.3%) like a friend, loved one, or support group. Some people (11/461, 2.4%) heard about the program from multiple sources, including loved ones, health professionals, or breast cancer organizations *and* social media.

### Implementation and Maintenance: Program Fidelity and Costs

#### Fidelity

A total of 42,344 SMS text messages were sent from April 29, 2020, to May 10, 2021; 41,257 (97.43%) were delivered successfully, 1057 bounced (2.50%), and 30 (0.07%) were pending (ie, neither delivered nor bounced). In total, 62/838 (7.4%) participants opted out; 48 participants opted out by replying “STOP” and 14 sent an SMS text message reply requesting to opt-out. Reasons for opt-outs are summarized in Figure 1. The results of the independent *t* test found that the mean age of participants who opted out (57.1 years, SD 11.0 years) did not differ significantly from those who remailed enrolled (*P*=.15). Most participants who opted out were White (54/61, 89%), finished treatment >3 years ago (26/61, 43%), and lived in Major Cities (45/61, 74%), in Australia’s least disadvantaged areas (IRSAD quintile 5; 27/61, 44%). The Fisher exact test did not indicate a significant association between ethnicity (*P*=.32), time since finishing treatment (*P*=.15), or remoteness (*P*=.87) and opting out. However, there was a significant association between IRSAD (*P=*.03) and opting out, in which participants were less likely to opt-out if they resided in Australia’s most disadvantaged areas: quintile 1 (3/61, 5%) or quintile 2 (5/61, 8%) relative to participants residing in quintile 3 (15/61, 25%), quintile 4 (11/61, 18%), or quintile 5 (27/61, 44%).

#### Cost

The direct program costs of promoting, delivering, and staff monitoring of EMPOWER-SMS COVID-19 to 841 participants was Aus $12,948.87 or Aus $15.40 per participant. Costs included social media (Aus $2853.31) and radio (Aus $2500) advertising, direct SMS text message costs (Aus $3387.52), and purchasing of automated SMS text message delivery software (Aus $1287). The estimated costs for staff to monitor incoming SMS text messages and send minimal replies (approximately 75 minutes per week for 54 weeks; 67.5 hours of work) were Aus $2921.04 based on a base salary of Aus $78,760 or Aus $43.27 per hour. In total, staff members sent 14 SMS text message replies, responding to direct questions about the study (4/14, 29%), general health questions where participants were directed to speak to their health care professional (4/14, 29%), confirming other requests (5/14, 36%), or requesting to receive the program again (1/14, 7%). Importantly, there were no urgent SMS text messages relating to safety or health-related issues.

### Adaptations

During the study period, no adaptations were made to the intervention. However, based on the qualitative data regarding patient engagement and satisfaction, the SMS text messages have since been adapted and personalized for future use. For example, additional weblinks, resources, and breast-cancer specific health tips have been added and a personalized option has been introduced for people taking endocrine therapy medication to receive specific SMS text messages about managing side effects and medication adherence. Other adaptations could include adding customizations, such as selecting the number of SMS text messages per week or timing of SMS text message delivery.

## Discussion

### Principal Findings

During the COVID-19 lockdowns, our team rapidly adapted the EMPOWER-SMS text message intervention [31,33] to deliver widespread COVID-19–related health support to breast cancer survivors. To our knowledge, this is the first study of its kind among people with lived experience of breast cancer. A variety of health professionals and organizations, including prominent Australian not-for-profit breast cancer organizations (eg, Breast Cancer Network Australia), supported the promotion of EMPOWER-SMS COVID-19 through social media, emails, newsletters, and word of mouth. Promotions led to rapid recruitment of 841 socioeconomically diverse breast cancer survivors across Australia within 9 months. The program was delivered as planned, with minimal “bounced” SMS text messages or opt-outs. The program required minimal cost or staff time and participants perceived EMPOWER-SMS COVID-19 to be acceptable, useful, and motivating for behavior change. Moreover, participants felt ongoing support was important for their recovery and helped them feel less alone, especially during the lockdowns. However, some participants would like the autonomy to choose program features that suit their preferences, schedules, and lifestyle, and would like specific SMS text message programs for patients during, directly after, and years after breast cancer treatment.

### Comparison With Prior Work

The results from our self-enrollment data suggest that EMPOWER-SMS COVID-19 had a broad reach, with participants registering from diverse ages, socioeconomic backgrounds, urban and rural communities, and times since finishing treatment. Similar results were seen in the Text4Hope program, which was launched in Canada in March 2020 to support people’s mental health during COVID-19 lockdowns. Within 1 week, 32,805 people subscribed; the majority were White, females, and aged 26-60 years [28]. Research shows that patients with other chronic illnesses residing in rural and remote communities around the world also desire delivery of health information and support via SMS text messages to improve access to health information and reduce barriers to in-person support, such as time and distance [38-40]. Our study used various resource-light strategies, including social media and familiar breast cancer organizations’ e-newsletters, which may have contributed to the rapid enrollment rate [41,42], as electronic communication has become ubiquitous. Moreover, research has found that recruiting participants through various strategies, including targeted social media advertisements, can increase participation of people from underserved populations [43]. Our data supported these findings, with one-third of participants (251/838) living in Australia’s most disadvantaged areas (IRSAD quintiles 1 and 2). These participants were also the least likely to opt-out of the intervention.

Interestingly, 35.8% (301/841) of participants had finished treatment more than 3 years ago, suggesting an ongoing desire for support for health management after breast cancer. Although not statistically significant, a large proportion of participants who opted out of the study were in this long-term survivorship phase. Qualitative results revealed that these participants wanted to move on from their breast cancer experience and no longer wanted breast cancer–specific support, but instead wanted to receive general health and wellness support. This result mirrors findings from a cohort study of 1083 long-term cancer survivors (N=1083; mean 47 months following diagnosis) who reported ongoing symptoms of anxiety and depression and that being insufficiently informed about health support options was a major unmet need [44]. There is some evidence that fear of recurrence and total unmet needs among long-term survivors could be improved using a health management support website compared with usual care [45]. Moreover, our previous qualitative feedback from patients within 18 months postactive treatment revealed that connecting patients to science-based websites and other community-based health resources using links in SMS text messages was useful [24]. This SMS text message intervention may therefore be an acceptable and useful way to deliver evidence-based health support to long-term survivors directly *and* link them to websites and resources.

Our current study also found that EMPOWER-SMS COVID-19 was delivered as planned with minimal cost (Aus $15.34/participant for 3 months, including staff time). The opt-out rate was low (62/838, 7.4%) and 97.43% (41,257/42,344) of SMS text messages were delivered successfully, indicating that the remaining participants received the entire EMPOWER-SMS COVID-19 program (49 SMS text messages/person). The low opt-out rates may have been due to participants’ perceptions that EMPOWER-SMS COVID-19 was highly acceptable, supportive, engaging, and useful for health management. SMS text message interventions have also been found highly acceptable among patients with psychiatric or mental health disorders [46,47], cardiovascular diseases [18-20,48], and other cancers [49]. Moreover, participants were engaged with the program, frequently reporting that they enjoyed the weblinks, and 59.9% (511/852) of participants replied to at least one SMS text message. Importantly, most participant replies were to answer our direct question or say “thanks,” with no urgent safety or health-related issues requiring a reply from the health counselor, which is consistent with previous research [20]. This form of light-touch support without significant increase to staff workload is important for reducing adoption, implementation, and maintenance barriers among health professionals.

It is also important to remember that program improvement is an iterative process and adaptations may be needed to improve and maintain participant engagement and satisfaction over time. Although no adaptations were made to the EMPOWER-SMS COVID-19 SMS text messages during the study, these findings and results from the original EMPOWER-SMS program [24,25] have been used to make adaptations for future renditions of the program, including additional weblinks, personalizations for people taking endocrine therapy medications, and more breast cancer–specific information. Researchers aiming to use a similar low-cost, scalable SMS text messaging strategy for patients with other conditions should consider co-designing SMS text message content and delivery (eg, timing, frequency) with their end users. Similar SMS text message programs have been co-designed to support adolescent health as well as people with endometriosis and other chronic diseases, to identify and address their unmet health care needs [50-53]. Moreover, providing opportunities for ongoing feedback through SMS text message replies or surveys enables immediate adaptations to SMS text messages, including editing weblinks or SMS text message content, or postintervention adaptations [20,24,54]. This study also used a publicly available automated SMS text message delivery software to send the intervention to participants. However, different health care settings use various software systems to deliver SMS text messages to patients. Adaptations made from the current program will be used to test the implementation of new EMPOWER-SMS programs into usual care in different settings within Australia, including the Westmead Breast Cancer Institute, a public hospital in Sydney, and in primary care practices nationally (National Health and Medical Research Council of Australia Emerging Leader Fellowship Investigator Grant ID 2017575). Further evaluation of barriers and enablers to adoption, implementation, and maintenance, and necessary adaptations from health care professionals working in different settings will be critical for future scale-up.

### Limitations

Although the SMS text message delivery data show that participants likely received the entire EMPOWER-SMS COVID-19 program, the user feedback survey had a 53.4% (449/841) response rate. Therefore, feedback may not be representative of all participants. However, this response rate is typical or higher than previous online surveys (20%-51% response rates) [55,56] and was achieved despite many people experiencing survey fatigue during the COVID-19 pandemic [57,58]. Of those who responded, many provided feedback that the SMS text messages motivated healthy eating and physical activity; however, effectiveness for improving these health outcomes was not evaluated. Although SMS text message interventions have been found to be effective for improving healthy eating and physical activity for patients with other chronic diseases [18,19,59,60], further research is needed to evaluate the effectiveness of SMS text messages to improve health outcomes for this population (breast cancer survivors). Finally, although this study found high participant acceptability and low costs to deliver the program, evaluating strategies for long-term program maintenance, including funding, will be central for successful population-level program integration.

### Conclusions

EMPOWER-SMS COVID-19 was rapidly implemented, had a broad reach, with high retention, acceptability, and engagement among diverse participants. Several factors influencing engagement with the program arose, including (1) feeling supported and less alone, (2) motivation and reassurance for health self-management, (3) the variety of information, (4) weblinks to information and resources, and (5) the option to save the SMS text messages. Moreover, the program was low cost, which may facilitate future implementation. However, further research is needed to evaluate the effectiveness for improving health outcomes, adoption, and implementation enablers and barriers for health professionals and strategies for long-term program maintenance.

## Appendix

Multimedia Appendix 1 TIDieR (Template for Intervention Description and Replication) checklist.

Multimedia Appendix 2 Example SMS text messages.

## Abbreviations

e-consent: electronic consent
IRSAD: Index of Relative Socio-economic Advantage and Disadvantage
RE-AIM: Reach, Effectiveness, Adoption, Implementation, and Maintenance
REDCap: Research Electronic Data Capture
TIDieR: Template for Intervention Description and Replication

## Availability of Data and Materials

The data sets used or analyzed during this study are available from the corresponding author on reasonable request.
