# Supplementary material for: A National Health and Wellness SMS Text Message Program for Breast Cancer Survivors During COVID-19 (EMPOWER-SMS COVID-19): Mixed Methods Evaluation Using the RE-AIM Framework
Source: J Med Internet Res. 2023 Jul 25;25:e45164. doi: 10.2196/45164 (PMC10410388; doi:10.2196/45164)
Supplement: Multimedia Appendix 2 [file jmir_v25i1e45164_app2.docx]

**Supplementary Table 1. Example text messages from EMPOWER-SMS COVID-19**

| **Message Topic** | **Example Text Message** |
| --- | --- |
| Social and emotional support | Hi [pref_name]^a^, mental health is closely linked to physical health. Today is a new opportunity to do something nice for yourself. Why not plan to do something you enjoy this week? |
| Physical activity/ Healthy eating | Hi [pref_name]^a^, need some ideas of physical activities you can do inside? Walking, dancing, stairs and yoga can all be done in the comfort of your home! |
| General breast cancer information | Not all information on the internet is reliable. Breast Cancer Network Australia shares websites with accurate breast cancer information: tinyurl.com/BCSites |
| COVID-19 advice | Hi [pref_name]^a^, if you have any questions about breast cancer and COVID-19, the BCNA Helpline 1800 500 258 is available 9am-5pm, 7 days per week |

^a^ [pref_name]: Participants preferred name entered at program enrolment.
